# Predicting Methane Concentration in Longwall Regions Using Artificial Neural Networks

**DOI:** 10.3390/ijerph16081406

**Published:** 2019-04-18

**Authors:** Magdalena Tutak, Jarosław Brodny

**Affiliations:** 1 Faculty of Mining and Geology, Silesian University of Technology, 44-100 Gliwice, Poland; 2 Faculty of Organization and Management, Silesian University of Technology, 44-100 Gliwice, Poland; jaroslaw.brodny@polsl.pl

**Keywords:** methane hazard, methane concentration, forecasting, in-situ measurements, artificial neural networks

## Abstract

Methane, which is released during mining exploitation, represents a serious threat to this process. This is because the gas may ignite or cause an explosion. Both of these phenomena are extremely dangerous. High levels of methane concentration in mine headings disrupt mining operations and cause the risk of fire or explosion. Therefore, it is necessary to monitor and predict its concentration in the areas of ongoing mining exploitation. The paper presents the results of tests performed to improve work safety. The article presents the methodology of using artificial neural networks for predicting methane concentration values in one mining area. The objective of the paper is to develop an effective method for forecasting methane concentration in the mining industry. The application of neural networks for this purpose represents one of the first attempts in this respect. The method developed makes use of direct methane concentration values measured by a system of sensors located in the exploitation area. The forecasting model was built on the basis of a Multilayer Perceptron (MLP) network. The corresponding calculations were performed using a three-layered network with non-linear activation functions. The results obtained in the form of methane concentration prediction demonstrated minor errors in relation to the recorded values of this concentration. This offers an opportunity for a broader application of intelligent systems for effective prediction of mining hazards.

## 1. Introduction

Underground hard coal mining is exposed to a series of natural hazards, one of the most dangerous being the methane hazard [1,2,3,4,5,6,7]. It is caused by methane release during the process of hard coal exploitation. When reaching certain concentration levels, this gas becomes flammable and explosive [1,3,7]. 

Methane explosion or ignition in an underground mine heading poses a great risk, which has been observed to have increased in recent years. This results from the exploitation of coal beds of high methane-bearing capacity, the great depth of mining operations, and high concentration of extraction. Underground mining exploitation in the world has led to numerous disasters related to methane ignition and explosion, see Table 1. 

It should be stressed that underground hard coal exploitation is and will be inextricably linked with the methane hazard. This is due to the formation of methane which occurred simultaneously with the formation of coal beds. Therefore, the exploitation of these beds will naturally cause this gas to release, which is—in this case—referred to as coal-mine methane or coal-bed methane [5,8].

**Figure 1:**
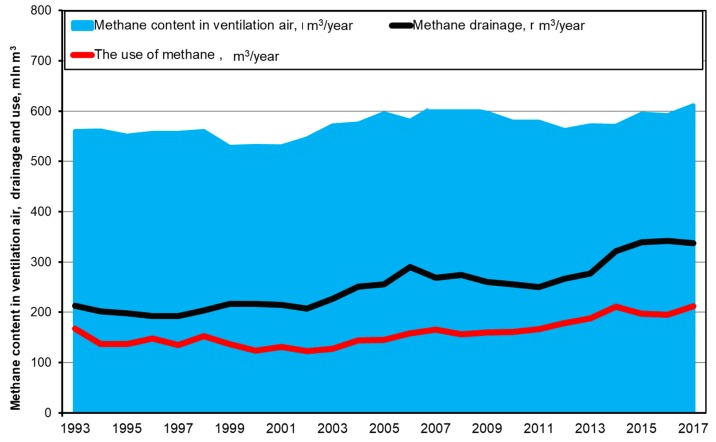
The amount of methane released into the atmosphere in Polish hard coal mines.

Over the last two decades in Poland, despite the fact that hard coal production has been limited, the amount of methane released during mining exploitation has been on the rise, see Figure 1 [11]. This results from the exploitation of hard coal beds of high methane-bearing capacity. Unfortunately, forecasts indicate that, in the coming years, despite a further decline in hard coal production, the methane hazard will not decrease.

It should also be stressed that methane, besides posing a safety threat to coal mines, is one of the most harmful greenhouse gases and constitutes a major risk for the natural environment [9]. The present study will concern only its negative impact on the process of mining exploitation and the work safety of the crew.

Thus, a very important role is played by all the forecasting activities concerning the determination of methane concentration values that may occur in the area of ongoing exploitation. The effectiveness of such activities depends on the forecasting method applied as well as on the reliability and quality of the input data that constitute the basis for the corresponding analysis. Numerous methods can be used for assessing the methane hazard, including empirical [12,13,14], analytical [15,16], numerical [17,18,19,20], short-term forecasting [21] and hybrid [22,23,24]. These methods vary in terms of their effectiveness. In a number of cases, the results obtained are unsatisfactory and substantially different from the reality.

Currently, in most cases, the potential values for the volumetric quantity of methane released into mine headings are determined with an approximation based on exploitation forecasts. In recent years, the quantities of methane released into mine headings have been increasingly predicted by means of numerical methods [1,2,3,5,17,18,19,20,24,25,26], including computational fluid mechanics [1,2,5,17,18,19,20].

Recently, there have also been studies on the application of intelligent systems for predicting this hazard on the basis of automatic systems registering ventilation parameters in the headings at risk [3,24,25,26]. In [25], the authors presented a rule-based approach to methane concentration prediction. The approach is evaluated on a real-life data set acquired during a week in a coal mine. The results show the advantages of the method introduced (in terms of both prediction accuracy and knowledge extraction) in comparison to the standard approaches typically implemented in the analytical tools. A different study [26], on the other hand, presents an approach based on the Very Fast Decision Rules algorithm and its application to the prediction of alarm states resulting from methane hazard in coal mines.

In [3], the authors applied a neural-fuzzy system to predict the methane concentration in the region of a longwall. This paper presents the possibilities of using artificial intelligence for the construction of predictive models based on measurement data. The discussion also encompasses the fundamental problems concerning fuzzy logic, neural network and the adaptive neuro fuzzy inference system (ANFIS). The work also presents an example of how its interface can be used for predicting methane hazards in the region of a mined longwall. A predictive model was developed based on the real methane measurement data from this longwall.

Despite the limited number of papers concerning the application of intelligent methods to predict methane concentration, they are the methods which—in the authors’ opinion—should be used more extensively for this purpose. This is because these methods, compared to those currently used, can be decidedly more effective in improving the safety of mining exploitation. Besides their high reliability and effectiveness, they should also offer the possibility to undertake quick actions. They must allow for the preparation of a relatively reliable prognosis in a short time. This is because time plays a crucial role in the safety of exploitation in the case of mining operations carried out in the zone of a high methane hazard. The reason for this is that it allows for effective prevention measures. For the prognoses determined to be reliable, use was made of the data registered by the automatic gasometry system, while the analysis was performed using artificial neural networks. 

The solution proposed in the paper makes it possible to determine the methane concentration levels in a mine heading 15 min in advance. An essential element of the method developed is the fact that it is based on real-world measurements of ventilation parameter values in a mine by the sensors of an automatic gasometry system.

The method developed was verified in the actual region of mining exploitation. Direct measurement results of the ventilation parameters in in-situ conditions led to the development of an artificial neural network model, which was subsequently subjected to analysis. The results obtained were compared with the measurement results, obtaining satisfactory correlations. 

The results obtained are very promising and, in this context, give reason to hope that the methodology developed will find practical applications in the mining industry, thereby improving the safety and effectiveness of the exploitation process.

## 2. Materials and Methods 

### 2.1. Study Area

**Figure 2:**
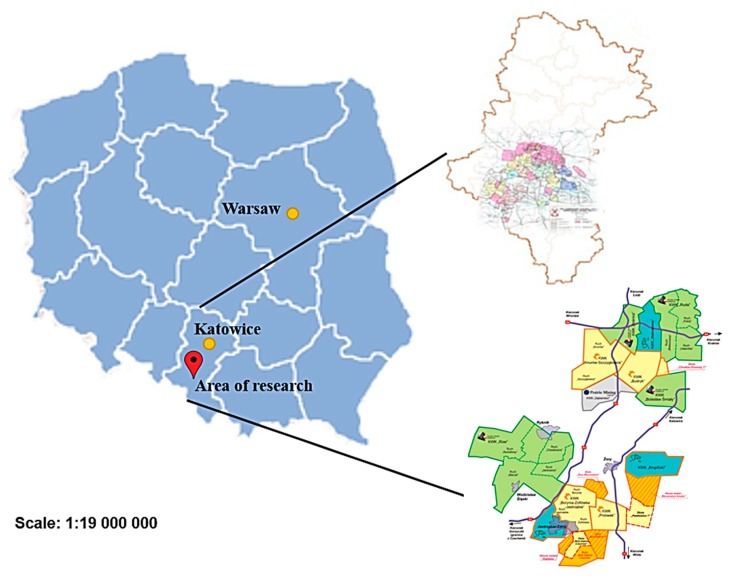
Location of the region where the tests were conducted, modified from [25].

The forecasting method developed was based on the results of methane concentration measurements at the inlet to and outlet from the longwall heading under analysis. The analyses were conducted for a real-world longwall in one of the mines of the Upper Silesian Coal Basin (GZW) area in Poland. The location of the region under analysis has been presented in Figure 2.

This mine is located in the southern part of GZW (Upper Silesian Coal Basin). Its exploitation area is 44.4 km^2^, whereas its extractable resources amount to 155.9 million tonnes of coal [27].

The longwall under analysis was exploited by means of a longitudinal system with a roof fall from the exploitation field borders. It is 216 m long, with a panel length of 1050 m, the thickness of the seam is 2.54 m, and the longitudinal inclination is 4.9° while the transverse inclination is 1.9° [28].

**Figure 3:**
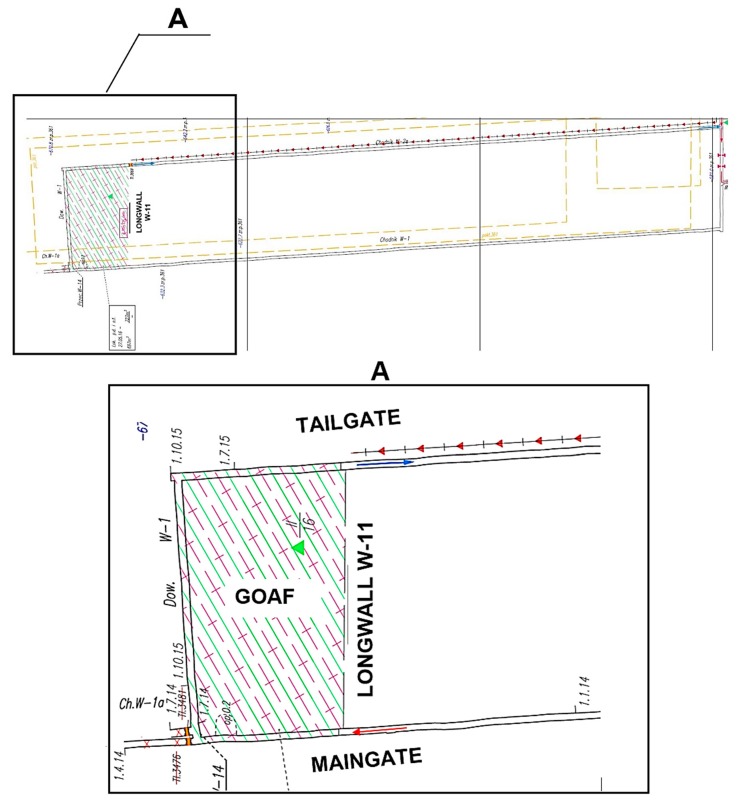
Location of the longwall under analysis, modified from [28].

The longwall is ventilated by means of the U-type system from the borders, with fresh air being supplied along the tailgate through an auxiliary air duct. The location of the longwall under analysis has been presented in Figure 3.

**Figure 4:**
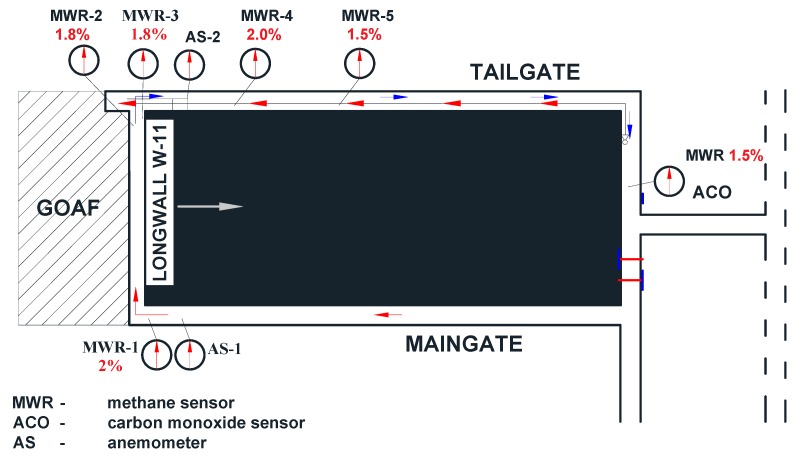
The distribution of sensors for measuring air parameters in the region of the longwall examined.

This wall was entirely covered by an automatic gasometry system equipped with methane sensors. The distribution of the sensors for measuring air parameters in the region of the longwall in question, along with the specification of alert thresholds, has been presented in Figure 4. The technical and ventilation parameters of the region under analysis have been summarized in Table 2.

While designing the mining exploitation process and developing the technical design specifications for the longwall, calculations were performed for the minimum volume output of fresh air that needs to be supplied to the longwall due to the methane hazard. This value amounted to at least 1100 m^3^/min. This stage also included the determination of the predicted methane amounts that will be released during exploitation. This value amounted to 12.36 m^3^ of CH_4_/min.

During exploitation, air was supplied to the longwall along the maingate at the average rate of 1000 m^3^/min., whereas the air supply rate along the tailgate with a built-in auxiliary air duct into the intersection area between this tailgate and the longwall was equal to approximately 141 m^3^/min.

### 2.2. Methods 

The evaluation of the methane concentration commonly is used in other fields. The purpose of the prediction was to determine the methane concentration in the measurement points located in the region under analysis and is presented in Figure 4.

Artificial neural networks, which have been intensively developed in recent years, constitute a universal approximation system that allows for reproducing multidimensional data sets and which is capable of learning and adapting to the changing environmental conditions, as well as generalising the acquired knowledge, thereby forming a system of artificial intelligence [29].

An artificial neural network represents a simplified scheme of the human brain. The basic constituent of this network is the processing element. It represents a specific model of real-life cells composing the nervous system, responsible for the processing and analysis of information in the human body. A real-life nerve cell can be treated as a biological system for processing information. The information introduced through the inputs (dendrites) is processed inside the cell. The processed signal is then transmitted to other cells through an axon. 

**Figure 5:**
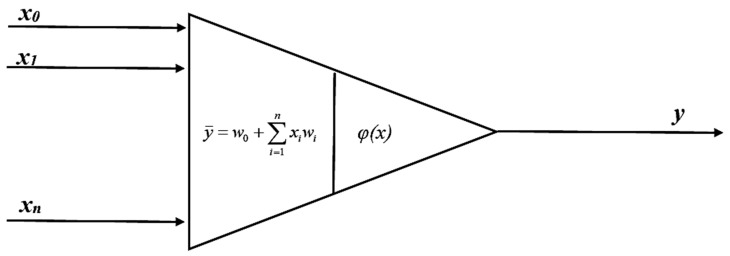
The basic model of a neuron, modified from [29].

Depending on its application, an artificial neural network is composed of a relevant number of elements having the capacity for processing information (neurons), which are combined with one another by means of connections with specific parameters (weights) that change during the learning process. The basic model of a neuron has been presented in Figure 5 [29].

The neuron shown in Figure 5 has *n* inputs *x_1_,*…., *x_n_*, one output *y* and a fixed value for each neuron independent of the input data, referred to as bias (*w_0_*) [30]. Input signals are processed based on the calculation of the weight sum of the inputs (including the bias for which the input value is always equal to 1), by their corresponding weights:

(1) $$\bar{y}=w_{0}+\sum_{i=1}^{n}w_{i}x_{i}$$

as well as the conversion of the resultant neuron stimulation value by the activation function, so as to obtain the following form of an output signal:

(2) $$y=\varphi \left(\bar{y}\right)$$

An activation function can be linear or non-linear. The most common activation functions include:

logistic function:

(3) $$\varphi \left(x\right)=\frac{1}{1+\exp\left(-\beta x\right)}$$

the hyperbolic tangent function:

(4) $$\varphi \left(x\right)=\frac{\exp\left(\beta x\right)-\exp\left(-\beta x\right)}{\exp\left(\beta x\right)+\exp\left(-\beta x\right)}$$

The creation of an artificial neural network requires a specific connection of the neurons. Therefore, the most common type of connection used is the unidirectional connection of neurons into layers, where the neurons between the successive layers interact and those within a single layer exhibit no interaction with each other. The particular layers are most commonly connected with one another on a “peer-to-peer” basis. Such a structure is referred to as the so-called Multilayer Perceptron (MLP). The number of layers may range from 2 to a dozen or so. Based on numerous studies, it was observed that a multilayer network (composed of more than the input layer, one hidden layer and the output layer) is mathematically equivalent to a network with one hidden layer, the input layer and the output layer. However, it was not possible to identify the number of neurons that should be possessed by the hidden layer. As a result, where a solution is being sought for a given problem using multilayer perceptrons, there is no need to build networks with a large number of hidden layers. Numerous works indicate that a single hidden layer is often sufficient, in accordance with the principle that the simpler the network, the better [31,32,33,34,35,36,37,38,39,40,41].

The concentration levels of methane in the region under analysis (in the measurement points) were predicted by means of a Multilayer Perceptron (MLP) network. An MLP network is one of the most commonly used networks for predicting various types of phenomena [41,42,43,44]. Gardner and Dorling provided a detailed description of the MLP network in the paper [41].

The main advantages of an MLP network, compared to other statistical models, is the fact that the network can “learn” practically any relationship between the input and output variables, without external intervention, which makes it suitable for predicting and controlling complex non-linear systems [45].

A typical MLP network consists of input, hidden and output layers. From the mathematical point of view, a neural network is a finite state automaton that processes the set of input (explanatory) variables *x* ∈ *R^N^* (*R^N^* is the space of real numbers) into a set of output (response) variables—*x* ∈ *R^N^* by means of a superposition of non-linear functions of a single variable and their linear combination [46].

The definition presented results from the Kolmogorov theorem, according to which each continuous function of multiple variables may be expressed by means of a superposition operation using only the function of two variables, namely [47]:

(5) $$f\left(x_{1},x_{2}\ldots ,x_{n}\right)=\sum_{q=1}^{2n+1}h_{q}\left[\sum_{p=1}^{n}\varphi_{q}^{p}\left(x_{p}\right)\right]$$

where the *h_q_ (u)* and $\varphi_{q}^{p}\left(x_{p}\right)$ functions are continuous, with $\varphi_{q}^{p}\left(x_{p}\right)$ not belonging to the selection of the *f* function.

The application of the Kolmogorov theorem made it possible to solve complex problems using a relatively simple MLP-type neural network.

In an MLP model, each *x_j_* variable from the input layer links with each neuron of the hidden layer through the connections with the weights *w_ij_*. These values are then summed up, resulting in the *s_i_* signal:

(6) $$s_{i}=\sum_{j=1}^{N_{k-1}}w_{ij}\cdot x_{j}$$

**Figure 6:**
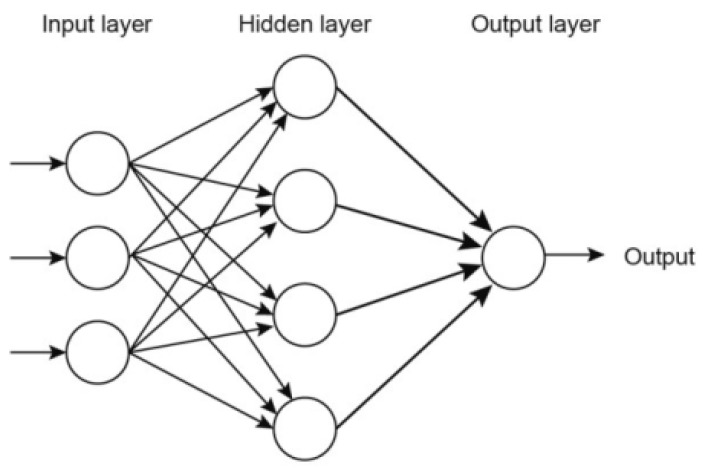
Typical Multilayer Perceptron (MLP) feed-forward Artificial Neutral Network Structure modified from [49].

The differentiable activation function inside the hidden layer converts this signal and sends the result *y_i_ (y_i_ = f(s_i_))* to the output layer. The number of neurons in the hidden layer is determined by a predefined approximation error, with the relationship between the number of neurons in the hidden layer and the approximation accuracy being directly proportionate. The weights of a given model are modified by means of an optimisation algorithm and the process is referred to as network learning. Since the error backpropagation algorithm uses the steepest descent method, this algorithm requires a large number of iterations, which may affect the speed of calculations. A typical structure of an MLP network has been presented in Figure 6 [33,34,35,48].

### 2.3. Preparation of Data from the Mine’s Gasometric System for the Forecasting of Methane Concentration Levels

The tests related to methane concenctration prediction were performed by means of the data recorded by a system of automatic methanometry in the mine, which consists of sensors for automatic registration of methane concentration levels in the measurement points (the sensor installation sites). In order to conduct the prediction process, the data acquired from this system were smoothed. This is due to the fact that automatic methanometers record methane concentration levels at a resolution of 0.1 CH_4_. These resolutions are sometimes too low and do not fully reflect the nature of the changing methane concentration levels in air. 

In the case at hand, the time courses of the changing methane concentration values recorded by the sensors of automatic methanometry were smoothed by means of the Holt’s method (which is one of the exponential smoothing techniques). This method involves smoothing of the time series by means of a moving average. The Holt’s model is described by means of the following equations [50]:

(7) $$F_{t}=\alpha x_{t}+\left(1-\alpha \right)\left(F_{t-1}+S_{t-1}\right)$$

(8) $$S_{t}=\beta \left(F_{t}-F_{t-1}\right)+\left(1-\beta \right)S_{t-1}$$

where *t* = *2, 3,…, n-1*; *F_t_* is the smoothed value of the time series; *S_t_* is the smoothed value of the trend gain (growth) per moment *t*; α, *β* are the smoothing parameters of the model, α, *β* ∈ [0,1].

The smoothing parameters are selected based on the criterion of the smallest average error from the expired forecasts [50]:

(9) $$s^{*}=\sqrt{\frac{1}{n}\sum_{t-1}^{n}{\left(y_{t}-y_{t}^{*}\left(\alpha ,\beta \right)\right)}^{2}}$$

**Figure 7:**
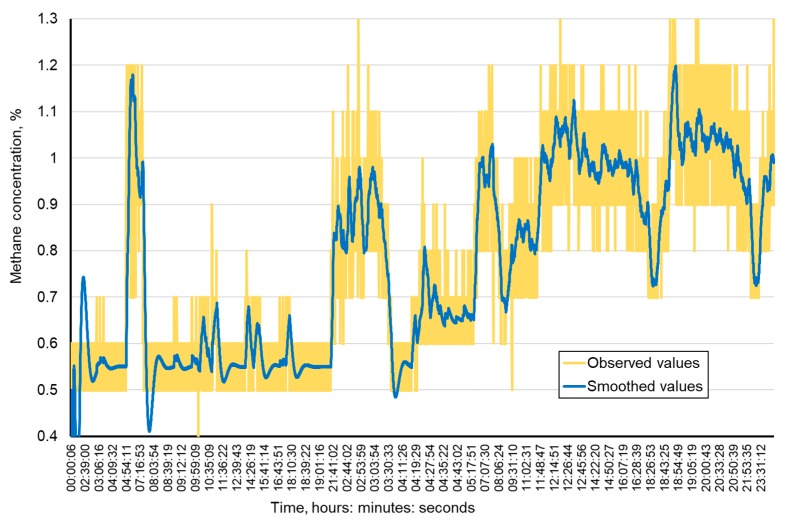
Sample smoothing of the time course of methane concentration in the airway.

An example of smoothed data coming from a methanometric sensor located at the outlet from the longwall, using the Holt’s method, has been presented in Figure 7.

**Figure 8:**
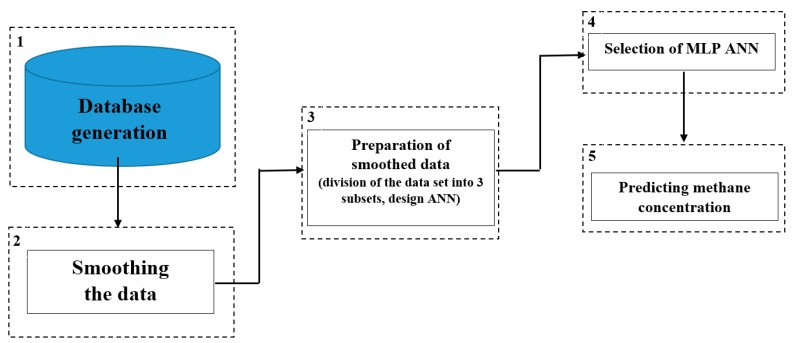
Methodological steps necessary to realize performing methane concentration.

The methodological steps, see Figure 8, required for predicting the methane concentration using artificial neural networks (ANN) techniques were:

1. Database generation;
2. Smoothing the data;
3. Dividing the data set (smoothed input variables) into training data, test data, and validation data, as well as designing the network and carrying out calculations;
4. Selection of the best network (the lowest error criterion);
5. Forecasting.

The smoothing of measurement data, whose total number amounted to 5403, was the first stage of the analysis. After being smoothed, the sets of data describing changes in the methane concentration values in the measurement points were divided into three subsets, namely the training data (70% of cases), the test data (15% of cases) and the validation data (15% of cases). The training data subset is used for network learning whereas the test data set is used for verifying the network training progress and the completion of the learning process before the occurrence of the network overtraining process. Network overtraining is a phenomenon that consists of excessive matching to a specific set of cases at the expense of losing the ability to generalise knowledge. The last set, namely the validation set, makes it possible to evaluate the network quality and compare different network variants applied to a concrete analysis. 

The prediction of the methane concentration in the area of the longwall was performed by means of the simplest possible network structure, composed of a single hidden layer. The hidden layer included up to eight neurons. From a number of available activation functions, the following have been verified in terms of their applicability to the prediction of methane concentration levels: The linear activation function, the hyperbolic tangent function, the logistic function and the exponential function. 

The calculation results for network learning based on the methane concentration values recorded within one day have been shown in Table 3, Table 4, Table 5 and Table 6. The results were sorted by network quality, expressed by the value of the linear correlation coefficient between the methane concentration value at the output from the network and the training data. The models were built using the ANN toolbox in the Statistica Software (version 13.3, TIBCO Software Inc., Palo Alto, CA, USA).

While selecting the model of a neural network, the lowest error criterion was applied. For this purpose, the following errors were determined from equations (10, 11 and 12):

- root mean square error (RMSE):
  (10) $$RMSE=\sqrt{\frac{1}{n}\sum_{i=1}^{n}{\left(X_{A,i}-X_{P,i}\right)}^{2}}$$
- mean absolute error (MAE):
  (11) $$MAE=\frac{1}{n}\sum_{i=1}^{n}\left(X_{A,i}-X_{{}_{P,i}}\right)$$
- mean absolute percentage error (MAPE):
  (12) $$MAPE=\frac{1}{n}\sum_{i=1}^{n}|\frac{X_{A,i}-X_{P,i}}{X_{A,i}}|$$
  where *X_A,i_* and *X_P,i_* represent the observed and predicted values.

A summary of the error values determined for selected models of neural networks (for network training) are presented in Table 7.

The MLP 1-3-1 network was selected for further analysis as the best of those presented in Table 3. The activation function adopted for the hidden neuron was the logistic function, while for the output neuron it was the linear function.

The MLP 1-8-1 network was selected for further analysis as the best of those presented in Table 4. The activation function adopted for hidden neurons was the logistic function, while for the output neuron it was the exponential function.

The MLP 1-2-1 network was selected for further analysis as the best of those presented in Table 5. The activation function adopted for hidden neurons was the hyperbolic tangent function, while for the output neuron it was the linear function.

The MLP 1-2-1 network was selected for further analysis as the best of those presented in Table 6. The activation function adopted for hidden neurons was the hyperbolic tangent function, while for the output neuron it was the linear function. 

## 3. Results and Discussion

The purpose of the calculations was to determine the effectiveness of the MLP network model developed, in terms of its capacity to forecast methane concentrations in the area of ongoing exploitation. The time horizon of the forecast was 15 min.

The calculations that were made for four measurement points served as the basis for determining the predicted methane concentration levels.

**Figure 9:**
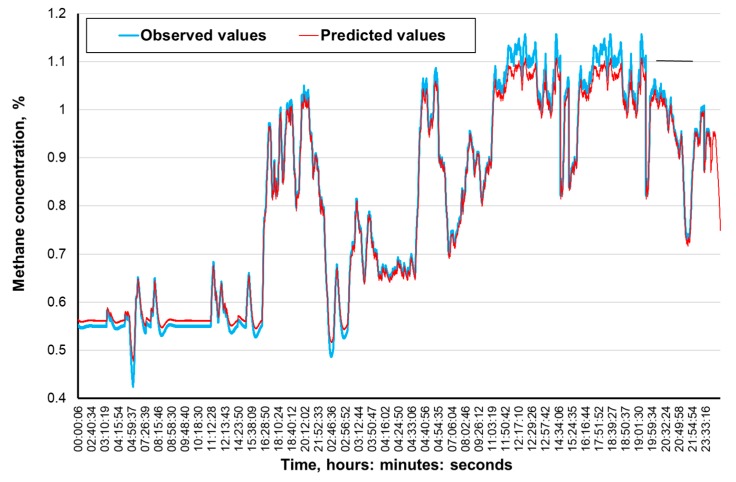
The time course of the registered and predicted methane concentration levels in the MRW-2 measurement point.

**Figure 10:**
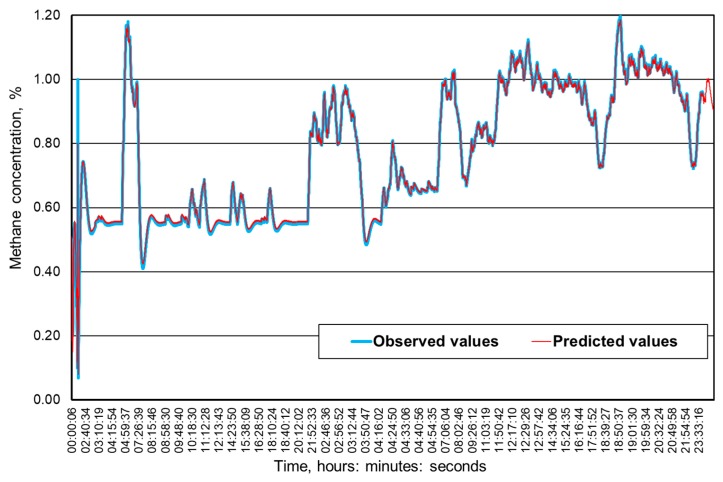
The time course of the registered and predicted methane concentration levels in the MRW-3 measurement point.

**Figure 11:**
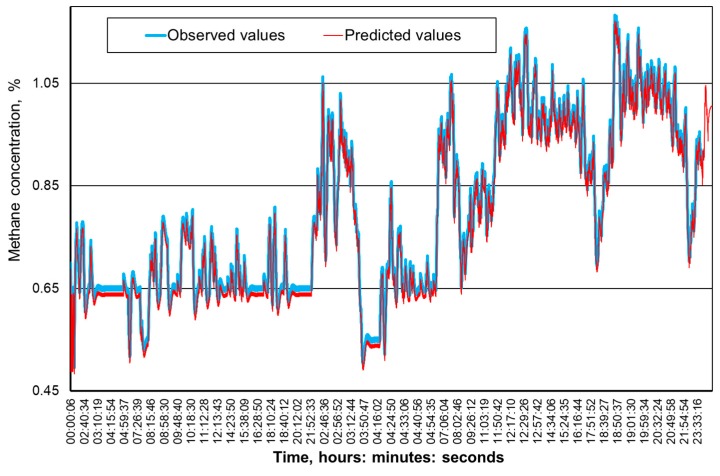
The time course of the registered and predicted methane concentration levels in the MRW-4 measurement point.

**Figure 12:**
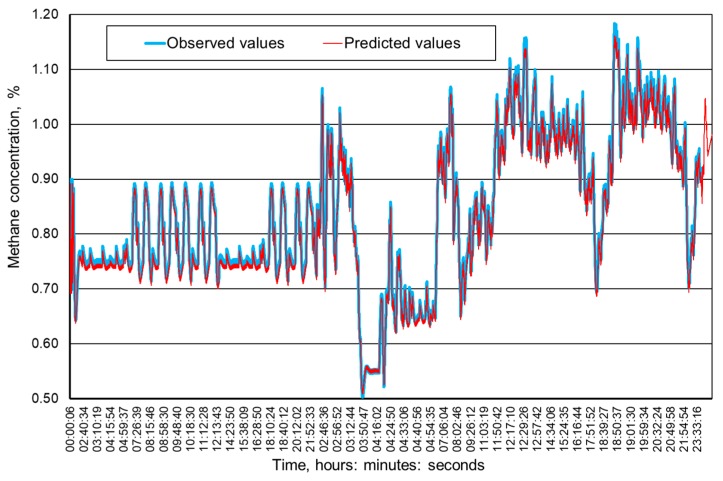
The time course of the registered and predicted methane concentration levels in the MRW-4 measurement point.

**Figure 13:**
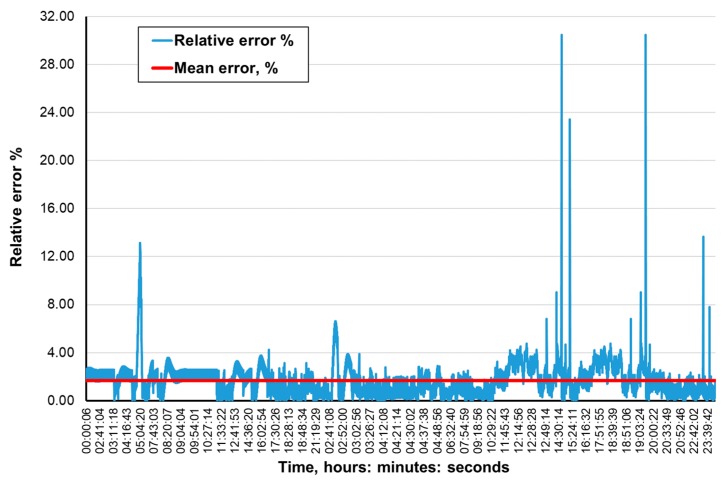
Percentage relative errors and mean forecasts of methane concentration levels in the MRW-2 measurement point.

**Figure 14:**
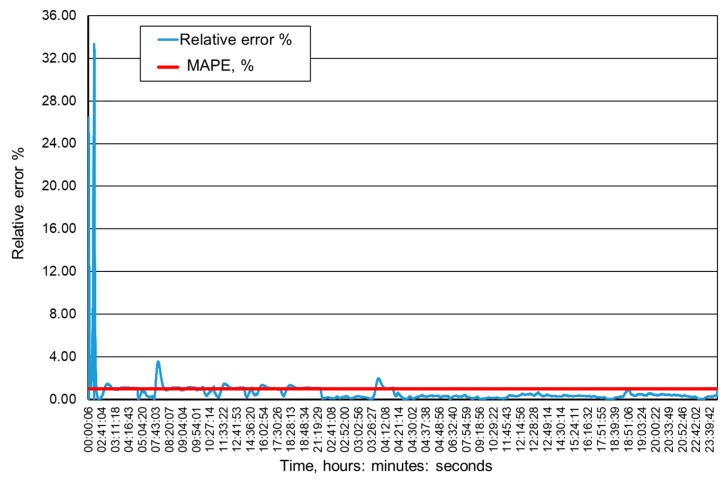
Percentage relative errors and mean forecasts of methane concentration levels in the MRW-3 measurement point.

**Figure 15:**
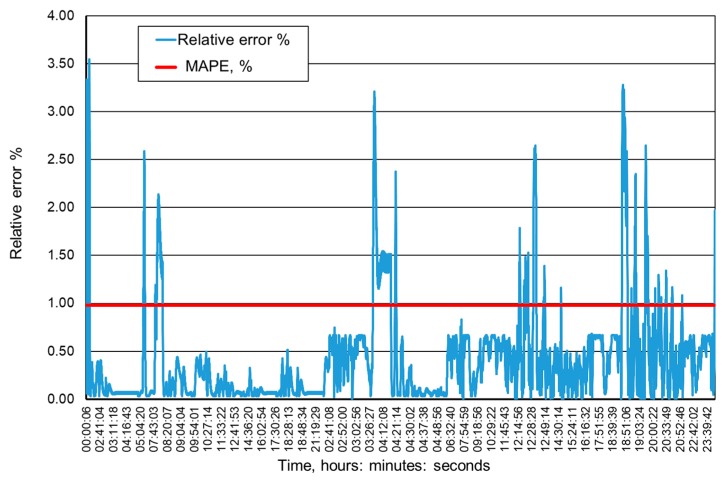
Percentage relative errors and mean forecasts of methane concentration levels in the MRW-4 measurement point.

**Figure 16:**
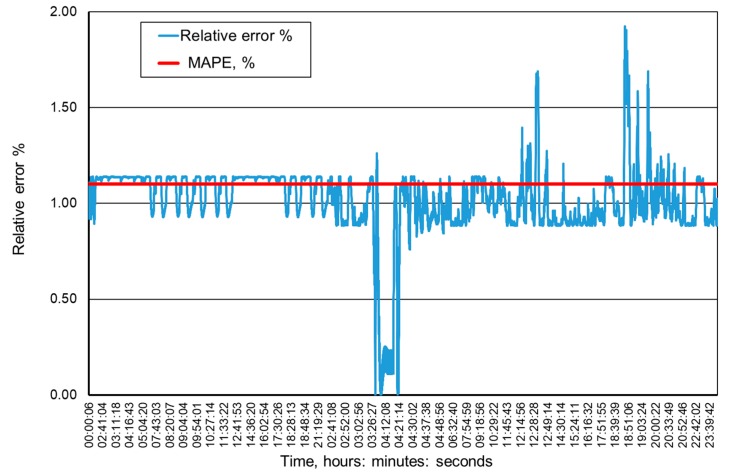
Percentage relative errors and mean forecasts of methane concentration levels in the MRW-5 measurement point.

Figure 9, Figure 10, Figure 11 and Figure 12 present the methane concentration levels recorded and calculated by a neural network in the measurement points for a single day. On the other hand, Figure 13, Figure 14, Figure 15 and Figure 16 present the forecast relative error and the mean error for those concentration levels in the particular measurement points. The relative error between the predicted and the registered value was calculated based on the following relationship (13):

(13) $$\mathrm{Relative}\;\mathrm{error}=|\frac{X_{A,i}-X_{P,i}}{X_{A,i_{}}}|\cdot 100\%$$

The neural networks used for predicting the methane concentration levels in the measurement points, despite their simple structures, allowed for a relatively accurate forecast of the methane concentration. The highest underestimation of the predicted methane concentration value amounts to 0.1% for the MRW3 measurement point.

It can be observed that the majority of errors fall within the range of 0.05% of the methane concentration, which should be considered as a good result.

The greatest forecast errors were observed for high concentrations of methane (over 0.8%) and during sudden increases in its value. Nonetheless, within the most common scope, the results obtained are satisfactory and acceptable to the service teams responsible for the mine’s ventilation safety. 

Therefore, it must be concluded that the MLP network architectures adopted make it possible to predict the methane concentration within the adopted time horizon with acceptable accuracy. At the same time, it seems reasonable to conduct further research in order to determine whether and possibly how much a change in this horizon would affect the accuracy of the results obtained.

**Figure 17:**
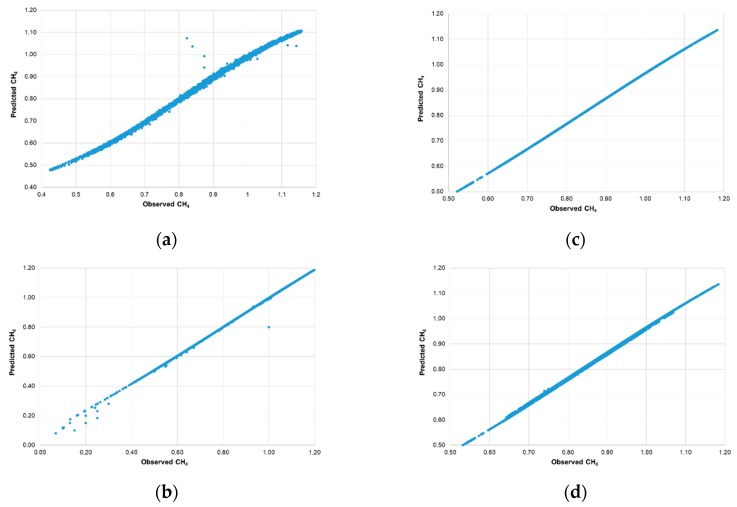
Predicted versus observed values CH_4_ ((**a**) sensor MRW-2, (**b**) sensor MRW-3, (**c**) sensor MRW-4, (**d**) sensor MRW-5).

The predicted values of the methane concentration and the observed values (registered by automatic methanometry sensors) are presented in Figure 17. 

From the data presented in Figure 17, it can be concluded that the highest dispersion is demonstrated by the data for the MRW-2 measurement point, while the lowest was demonstrated for the MRW-4 measurement point.

## 4. Conclusions

The methane hazard is one of the most dangerous phenomena in hard coal mining. Therefore, in order to maintain the continuity of the production process and the safety of work for the crew, it is necessary to undertake appropriate measures to ensure safe working conditions. 

The use of artificial neural networks allows for short-term prediction of the methane concentration in the area of ongoing exploitation, thereby improving work safety in this region. A forecast perspective amounting to 15 min is acceptable both in terms of work safety and effectiveness of the production process. It should be noted that the exceedance of the set point values of methane concentration in the measurements points leads, first and foremost, to discontinuation of the exploitation process and withdrawal of the crew from a given region. The supply voltage is switched off, the machines are brought to a standstill and the exploitation process is stopped until these values are reduced. This entails high economic costs. 

On the other hand, knowledge regarding future concentration levels makes it possible to take immediate corrective actions. Where an exceedance of the methane concentration levels is predicted to arise, it is possible to decrease the exploitation rate or increase the supply of fresh air to the headings. These active measures should reduce the concentration of methane and, consequently, ensure continuity of the exploitation process. They should also limit the possibility of critical concentrations that may lead to fire or explosion.

By analyzing the methodology developed and the results obtained, it may be concluded that a properly designed and trained neural network can be successfully used for predicting the methane concentration levels in the areas of ongoing mining exploitation. The results obtained demonstrate acceptable accuracy, and the forecasting time is short enough to allow for the undertaking of effective prevention measures in the event where dangerous methane concentrations occur. 

It should also be noted that the model presented works well with a relatively slow increase in the level of methane concentration. Sudden, unpredictable increases are more difficult to forecast (the forecast error is higher). Therefore, it seems reasonable to conduct further research on the development of this methodology.

Nevertheless, in the form presented herein, this methodology together with the model developed should constitute an essential tool for supporting the process of mining exploitation.

## Figures and Tables

**Table 1:** Underground coal mine disasters related to methane explosions and methane fires (examples) [9,10,11].

Year	Country	Coal Mine	Cause of Explosion	Fatalities
1887	Australia	Bulli	Ignition of gas and coal dust	81
1887	Belgium	La Boule, Borinage	Methane explosion	120
1914	Canada	Hillcrest	Methane and coal dust explosion	189
1914	Japan	Mitsubishi Hojyo	Methane explosion	687
1950	China	Henan	Methane explosion	187
1960	China	Henan	Methane and coal dust explosion	187
1972	Zimbabwe	Wankie Colliery Disaster	Methane and coal dust explosion	426
1991	China	Shanxi	Methane and coal dust explosion	147
2005	China	Liaoning	Methane explosion	214
2006	Poland	Halemba	Methane and coal dust explosion	23
2007	China	Hanxi	Methane explosion	105
2009	Poland	Wujek (Śląsk)	Methane and coal dust explosion	20
2009	China	Heilongjiang	Methane explosion	108
2014	Poland	Mysłowice-Wesoła	Methane explosion	5
2015	Poland	Sośnica	Methane fire	4
2016	Poland	Murcki-Staszic	Methane explosion	1

**Table 2:** Geometry and ventilation parameters of the test region.

Geometry and Ventilation Parameters	Values
Air emission rate (Q_1_)—maingate, m^3^/min	1100.00
Air emission rate (Q_2_)—air duct in tailgate, m^3^/min	141.0
Methane emission rate (absolute methane content—Q_CH4_), m^3^CH_4_/min	12.36
The height of longwall, m	3.5
The length of longwall, m	116.0
The width of longwall, m	3.5
The width of longwall galleries, m	4.0
The height of longwall galleries, m	3.5
The length of the unliquidated part of the airway behind the caving line of the longwall, m	4.0

**Table 3:** The learning results of the artificial neural networks included in the tests for Sensor 2 (MRW-2).

Network Structure	Correlation Coefficient			Matching Error			Activation Function—Neurons	
	Set			Set			Hidden	Output
	Training	Test	Validation	Training	Test	Validation		
MLP 1-5-1	0.99861	0.99725	0.99896	0.020398	0.020601	0.020035	logistic	logistic
MLP 1-3-1	0.99934	0.99786	0.99970	0.00002987	0.0000968	0.0000130	linear	linear
MLP 1-6-1	0.99879	0.99742	0.99917	0.0001570	0.0002180	0.00013725	logistic	linear
MLP 1-2-1	0.99924	0.99781	0.99961	0.00003421	0.00009938	0.00001723	hyperbolic tangent	linear
MLP 1-2-1	0.99934	0.99786	0.99970	0.000029725	0.00009698	0.000012916	linear	linear

**Table 4:** The learning results of the artificial neural networks included in the tests for Sensor 3 (MRW-3).

Network Structure	Correlation Coefficient			Matching Error			Activation Function—Neurons	
	Set			Set			Hidden	Output
	Training	Test	Validation	Training	Test	Validation		
MLP 1-4-1	0.996781	0.996866	0.996834	0.000093	0.000094	0.000089	hyperbolic tangent	exponential
MLP 1-8-1	0.997406	0.997327	0.997408	0.000070	0.000074	0.000068	logistic	exponential
MLP 1-8-1	0.997049	0.997127	0.997132	0.000082	0.000082	0.000076	hyperbolic tangent	linear
MLP 1-6-1	0.997406	0.997327	0.997408	0.000070	0.000074	0.000067	linear	linear
MLP 1-6-1	0.996996	0.996985	0.997105	0.000081	0.000084	0.000075	linear	linear

**Table 5:** The learning results of the artificial neural networks included in the tests for Sensor 4 (MRW-4).

Network Structure	Correlation Coefficient			Matching Error			Activation Function—Neurons	
	Set			Set			Hidden	Output
	Training	Test	Validation	Training	Test	Validation		
MLP 1-7-1	0.99678	0.99686	0.99683	0.000093	0.000094	0.000089	hyperbolic tangent	exponential
MLP 1-3-1	0.99740	0.99732	0.9974	0.000070	0.000074	0.000067	linear	linear
MLP 1-2-1	0.99704	0.99712	0.99713	0.0000815	0.000082	0.000076	hyperbolic tangent	linear
MLP 1-2-1	0.99740	0.99732	0.99740	0.000070	0.000074	0.0000674	linear	linear
MLP 1-3-1	0.99699	0.99698	0.997104	0.000081	0.000083	0.000075	logistic	sine

**Table 6:** The learning results of the artificial neural networks included in the tests for Sensor 5 (MRW-5).

Network Structure	Correlation Coefficient			Matching Error			Activation Function—Neurons	
	Set			Set			Hidden	Output
	Training	Test	Validation	Training	Test	Validation		
MLP 1-2-1	0.99566	0.99609	0.99579	0.00008085	0.000073	0.000073	hyperbolic tangent	linear
MLP 1-6-1	0.99560	0.99606	0.99574	0.00008197	0.000074	0.000074	linear	linear
MLP 1-3-1	0.99575	0.99614	0.99582	0.00007990	0.000073	0.000074	linear	linear
MLP 1-2-1	0.99575	0.99614	0.99582	0.00007911	0.000072	0.000073	linear	linear
MLP 1-7-1	0.995022	0.99552	0.99523	0.00011079	0.000101	0.000099	hyperbolic tangent	exponential

**Table 7:** Summary of errors for selected neural networks.

Neutral Network Structure (Sensor)	RMSE (Root Mean Square Error)			MAE (Mean Absolute Error)			MAPE, % (Mean Absolute Percentage Error)		
	Training	Test	Validation	Training	Test	Validation	Training	Test	Validation
MLP 1-3-1 (MRW-2)	0.0181	0.0358	0.0208	0.0135	0.0266	0.0147	1.669	3.088	1.919
MLP 1-8-1 (MRW-3)	0.0082	0.0162	0.0094	0.0079	0.0154	0.0085	0.965	1.737	1.085
MLP 1- 2-1 (MRW-4)	0.0075	0.0149	0.0086	0.0062	0.0122	0.0067	0.854	1.544	0.956
MLP 1-2-1 (MRW-5)	0.0093	0.0184	0.0107	0.0085	0.0167	0.0092	1.121	1.862	1.122
